# Valorization of coir peat in green extraction of valuable metals from spent lithium-ion batteries

**DOI:** 10.1371/journal.pone.0326867

**Published:** 2025-07-01

**Authors:** Sadaf Fatima, Muhammad Kaleem Khosa, Awal Noor, Sadaf Qayyum

**Affiliations:** 1 Department of Chemistry, Government College University Faisalabad, Faisalabad, Pakistan; 2 Department of Chemistry, College of Science, King Faisal University, Al–Hassa, Saudi Arabia; 3 Department of Basic Sciences, Preparatory Year, King Faisal University, Al–Hassa, Saudi Arabia; University of Technology and Applied Sciences - Salalah, OMAN

## Abstract

This research investigates an eco-friendly hydrometallurgical process for extracting valuable metals such as Li, Co, Ni, and Mn from spent lithium-ion batteries using biodegradable mixed organic acids, supported by coir peat as a natural reductant. Optimal leaching conditions (slurry density: 20 g/L, temperature: 55 °C, time: 55 minutes, stirring speed: 460 rpm, acid concentration: 50:50 mM ascorbic acid/citric acid) achieved metal efficiencies up to 85%. Incorporation of coir peat further enhanced the leaching efficiencies 98% for Li, 84.6% for Co, 85.6% for Ni, and 79.8% for Mn. Kinetic modeling revealed a chemically controlled dissolution process, with apparent activation energies of 43 kJ/mol (Li), 68 kJ/mol (Co), 47.8 kJ/mol (Ni), and 46 kJ/mol (Mn). Characterization through SEM confirms the morphological changes from spherical to uneven particle surfaces, FT-IR and XRD confirms the structural transformation of of cathode material before and after leaching, and UV-Vis spectroscopy showed the Co-complexes, Mn-complexes, and Ni-complexes at peaks around 380nm, 425 nm to 450 nm respectively due to reduction of metal complexes. The finding highlights the potential of biodegradable reagent and agro-waste material in developing sustainable battery recycling methods.

## Introduction

As the world transitions to produce renewable and less carbon energy, the supply of valuable metals will increase. The main components of lithium–ion batteries (LIBs) are valuable metals such as lithium, cobalt, nickel, and manganese [1]. Therefore, there is a constant and increasing need to make sure a consistent and efficient supply of metal components to support the long–term expansion of different electric vehicle (EV) and energy storage regions [2]. Various techniques, like pyro-metallurgy and bio–metallurgy, were used for the recovery purpose of valuable metals, while in hydro-metallurgical processes, metal ions are often dissolved using acidic reagents before being recovered through solvent extraction method and then separated by selective precipitation. The mixed acidic leaching system is an essential part of entire hydro–metallurgical processes [3]. The traditional acid leaching process typically uses a mixture of inorganic acid and H_2_O_2_ to recover valuable metals, which can contaminate the whole environment and provide potential hazards. As a result, further studies are needed for the leaching process using aqueous organic reagents that are both eco–friendly and safe [4].

The leaching of valuable metals from wasted LIBs is often achieved with commonly used organic acids (such as ascorbic acid, citric acid, succinic acid, malic acid, and oxalic acid) mainly due to their high efficiency and lack of contamination instead of inorganic acids [5]. Reducing agents like H_2_O_2_, NaHSO_3_, and organic reductants, are required to facilitate the process of leaching that involves the reduction of metals from high to low valence states in the cathode material [6]. However, to minimize environmental impact, it is crucial to identify a leaching system that has minimal use of acid and operates under mild conditions to achieve high leaching yield [7]. Chemical reducing agents can pollute the environment; therefore, selection of sustainable and non–chemical reducing agents could prove a better choice.

Adopting such environment friendly process, the cathode waste from the LIBs was placed in a ball mill tank along with a predetermined quantity of grape–skin powder (GS). Zhang group used the ball–mill (BM) medium to extract metals from the mechanically activated LIBs applying an H_3_Cit solution at a predetermined concentration to achieve maximum leaching of Li and Co metals (99% and 98%, respectively) [8]. Combine ball milling with a natural organic reductant, such as litchi peel, can also enhance the leaching rates of Li, Co, Mn, and Ni (99.21wt%, 96.52 wt%, 98.21 wt%, and 94.79 wt%, respectively) [9]. Cathode materials from used LIBs have been recycled using biomass on a large scale. In metal leaching, reducing agents such as bagasse [10], tea waste [11], molasses [12], lemon juice [13], and waste areca powder [14] have been utilized instead of H_2_O_2_. Coir peat, often known as coir, is derived from the fibrous husk found within coconuts. To make the coir, husks are steeped until the textile fibers can be separated [15]. It is composed of cellulose (43.44%), lignin (29.23%), hemicelluloses (8.50%), pectin 4%, and some correlated compounds (3.00%). Water–solubility (5.25%), low cost, high moisture content, low calorific value and availability, makes it less valuable as boiler fuel but offers advantages over traditional reducing agents [16]. Therefore, it remains of significant challenge to adopt new methodologies for the practical use of coir peat. Previously, coir peat has been used as chelating agent to remove metals such as Ni, Zn, Fe, and Pd from spent batteries [17]. The study suggested that some biomasses, like coir peat, can function as chelating agents and serve as alternative reductants to chemical ones to extract valuable metals like Ni, Co and Mn.

As an alternative to traditional reductant such as H_2_O_2_, we have tried to use a novel concept of using coir peat as a reductant, along with aqueous mixture of mild organic acids (ascorbic and citric acid) that were potentially used as reducing agents for leaching of spent cathodic material, resulting in an improvement in leaching performance. This method is eco-friendly, low cost. And sustainable and to our knowledge, it has not been reported before.

To report the matters of trash buildup as well as resource depletion, this paper recommends a unique idea for recycling end-of-life LIB waste: employing coir peat waste as a potential reducing agent. It is known as a “waste-for-waste” strategy when plant–based waste products are intentionally used to treat LIB trash. Heating of coir peat in an acidic environment allows them to reach their full potential as a sustainable reducing agent. It degrades 30% of cellulose and 70% of hemicellulose into aldehydes types reducing sugars. This study focuses on a novel and green process of using coir peat as a reductant for leaching of the cathode material. Most valuable metals are highly recyclable, which do not result in any loss of quality or change in their properties, which avoids using chemical reductants. Leaching mechanism, impact of various leachant constraints on the leaching kinetics, and efficiency of leaching procedure are examined. Atomic absorption spectroscopy (AAS) and inductively coupled plasma optical emission spectroscopy (ICP-OES) were applied to measure the total concentration of valuable metals. X-ray diffraction and scanning electron microscope (SEM) were used to examine the morphology of the metal–containing cathodic powder.

## Materials and methodology

Exhausted LIBs of mobiles and laptops (Li_x_Ni_y_Mn_z_CoO_2_, LiCoO_2_) were obtained from local market. Citric acid and ascorbic acid were obtained from Sigma–Aldrich® Solutions, St Louis, MO, USA 99% Extra pure, (C_6_H_8_O_6_).

### Analytical methods

Metal concentration in the leachant were measured by using an Atomic absorption spectrometer (AAS) model AA-7000 (Japan)/ Inductively coupled plasma optical emission spectrometer (ICP-OES), model Optima 8000 (PerkinElmer, USA). Scanning electron microscope (SEM-Tescan, Dortmund, Germany) was used to study the morphology. Powder-XRD (Raku) was used for phase identification. FT-IR spectroscopy (Billerica, USA) was used to confirm the changes in leaching environment during experiment.

## Experimental procedure

### Pre–treatment of sample

#### Treatment of the coir peat.

The coir peat was purchased from a nearby market. The coir peat was dried and ground, then soaked in water to expand and become fully hydrated, with particle sizes in the range of 2 mm to 8 mm that is suitable for effective leaching. After that, samples were dried in oven for three days at 50 °C to make sure that all the moisture was evaporated. Following drying, the coir peat was ground up and sieved over a 60 meshes screen (pore size–260 μm). This ensures good drainage and minimizes the retention of leachate, facilitating the effective extraction of valuable metals from black cathode powder. Since the coir peat has a large surface area and can adsorb certain valuable metals, and is thus useful in industrial use for recovering metals from used LIBs. This makes it a viable option for environmentally friendly metal recovery methods.

Coir’s hydroxyl cellulose group becomes oxidized to a carboxyl group, forming a weak cationic ion exchanger. At room temperature, 50 grams of coir fiber were added to 1 liter of a solution that contained 10 grams of 50% hydrogen peroxide and 1 gram of sodium hydroxide. Oxidation was maintained for two hours after that temperature was gradually increased to 60 °C to ensure drying and was then employed as an adsorbent [18].

#### Dismantling and separation.

It is necessary to dismantle the used LIBs manually to separate their components and recover the better active cathode powder. The LIBs were disassembled into different components, such as plastic/metallic containers, polymers, anode sheets, and cathode sheets. The cathode powder was held at 700 °C for about two hours in muffle furnace, causing spent cathode material and volatile contaminants to be easily eliminated from the aluminum and copper foils [19]. The resulting cathode powder was then subjected to ball mill grinding until it was transformed into a fine powder. After 30 minutes of grinding, the powder was reduced in size, which resulted in a high surface area to increase leaching efficiency. To prepare a uniform sample mixture for characterization and leaching analyses, this powder was then sieved through a 110–mesh screen.

#### Leaching experiment.

**Fig 1:**
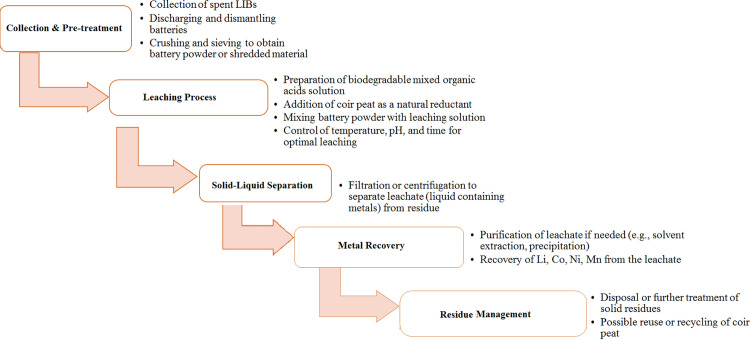
Flowsheet towards recycling of spent lithium–ion batteries (LIBs).

Fig 1 represents the flowsheet diagram that was used throughout the leaching process. 100 mL of aqua regia (HCl + HNO_3_) was used to digest 1 g of black powder (cathode powder) to get chemical analysis of LiCoO_2_ cathode. Inductively coupled plasma optical emission spectroscopy (ICP–OES) was used to determine the chemical composition of wasted battery cathode powder. The results showed 26 wt% cobalt, 17 wt% manganese, 6.0 wt% nickel, and 7 wt% lithium. Element analyses of black powder’s (LIBs cathode) and coir peat are displayed in Table 1.

**Table 1:** Element analyses of LIBs cathode (ICP measurements) and coir peat.

Cathode powder		Coir peat			
Metals	(mg/kg)	Coir peat	Percentage %	Analysis	Techniques
Al	728	Cellulose	24	Lignocellulosic composition (Dry basis)	Chemical Extraction
Ti	170	Hemicellulose	50		
Cu	560	Lignin	20		
S	1090	C	40	Elemental composition (Dry basis)	CHNS elemental analyzer
Fe	900	H	7		
Ca	170	O	48		
Mg	169	N	0.9		
Zn	269	Moisture	47	Oven Dry	

#### Leaching of cathode with mild organic acids and coir peat.

**Fig 2:**
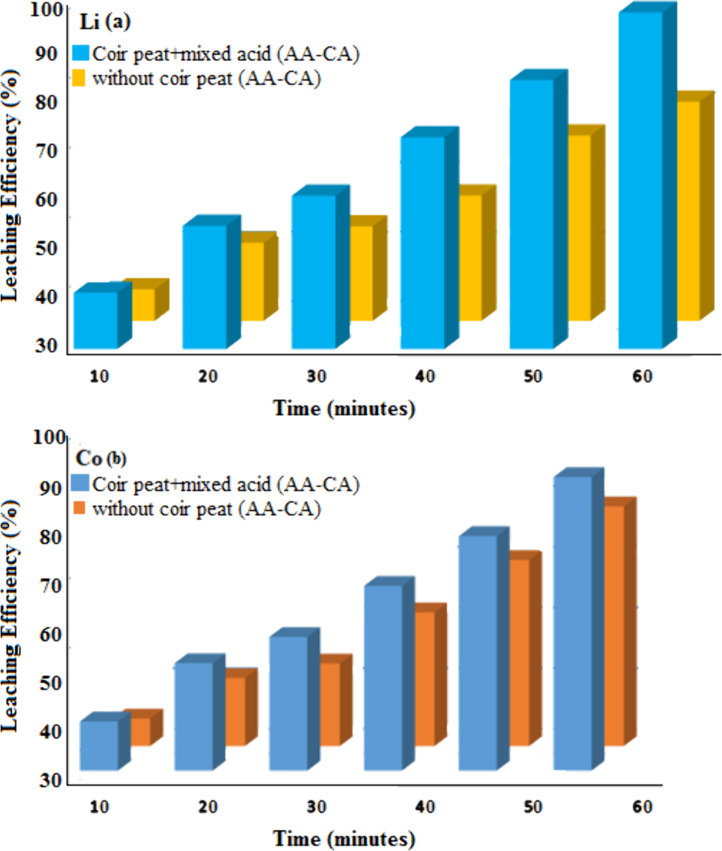
(a) Leaching efficiency of Li with mixed acids/coir peat and with only mixed acids (b) Leaching efficiency of Co with mixed acids/coir peat and with only mixed acids.

The group without coir peat acts as a control. Fig 2 shows the leaching efficiency of Li and Co from LiCoO_2_ cathode with and without the addition of coir peat with mixed organic acids (AA–CA). On the other hand, in comparison to the mixed acid (AA–CA) group, leaching efficiencies of Li and Co in lixiviant comprising coir peat obviously improved with time. Since the coir peat still retains trace quantities of metal prior to disposal, it would be possible to reuse it for additional cycles of metal adsorption following the hydrometallurgy process by extracting metal from used LIBs.

The experiments were conducted in batch mode. A 250 mL round–bottom flask equipped with a temperature control feature was utilized. A magnetic stirrer was attached to the flask to ensure uniformity (ranging from 200 to 600 rpm). A 4 g sample of black cathode powder was added into the equimolar ratio of ascorbic and citric acid (AA–CA) along with the desired concentration (1, 1.25, 1.5, 1.75, 2.00, 2.25, and 2.50 g/g) of coir peat. It was then heated until the required temperature (15–75 °C) for the leaching system, which was then subsequently fixed at the optimized temperature of 55 °C. The water bath was used to regulate the leaching temperature [20].

The progress of leaching treatment on leachate and reaction residue was evaluated by withdrawing sample specimens at regular intervals (15–75 minutes). The interval was afterwards fixed at the optimized time of 55 minutes and the sample was strained through Whatman 40 filter paper. The specific leaching efficiency of each metal for kinetic analysis was determined by taking a range of liquid samples (10–100 mL) at regular intervals during the leaching process. Deionized water was used to filter and purify the solution after it had leached. The filtrate of valuable metal content was examined using an atomic adsorption spectrophotometer (AAS). The following formula was used to calculate the leaching efficiency of valuable metals.


$$\alpha =\frac{m_{1}-m_{2}}{m_{3}}\times 100\;\%$$ (1)

Where m_1_ represents the initial mass of cathode, α indicates their leaching efficiency, m_2_ represents the filter residue weight, and m_3_ represents the metal concentration of cathode samples [21].

#### Chemistry of organic acids and coir peat for cathode powder leaching.

Citric acid has three carboxyl groups and is an ordinary weak organic acid. Adding one mole of citric acid to distilled water cannot produce three moles of H^+^. For leaching of LiCoO_2_, citric acid was used as a leaching reagent Equation (2–5).


$$H_{3}\text{Cit}=H_{2}{Cit}^{-}+{\text{H}}^{+}\text{k}a1\text{ =}7.5 \times 10^{-4}\mathrm{\backslash ]}$$ (2)

$$H_{2}{Cit}^{-}=H{Cit}^{2-}+{\text{ H}}^{+}k_{a^{2}}=1.8\times 10^{-5}\mathrm{\backslash ]}$$ (3)

$$H{Cit}^{2-}={Cit}^{3-}+{\text{ H}}^{+}k_{a3}=4.1\times 10^{-7}\mathrm{\backslash ]}$$ (4)

$$6H_{3}Cit+2LiCoO_{2}=2{Li}^{+}+2{Co}_{2}+6H_{2}{Cit}^{-}+2H_{2}O+O_{2}+H_{2}\mathrm{\backslash ]}$$ (5)

According to Equation (2), dissociation reaction is the most significant one having the largest K_a_ [22]. After leaching, the solution contains Li^+^, Co^2+^, Cit^3−^, HCit^2−^, and H_2_Cit^−^ which subsequently react to form complexes. Following expressions could represent possible coordination activities between metal–ligands ion:


$$3{Li}^{+}+{Cit}^{3-}=\left[{Li}_{3}{Cit}^{3-}\right]\mathrm{\backslash ]}$$ (6)

$$3{Co}_{2}+{2Cit}^{3-}=\left[{{Co}_{3}\left({Cit}^{3-}\right)}_{2}\right]\mathrm{\backslash ]}$$ (7)

**Fig 3:**
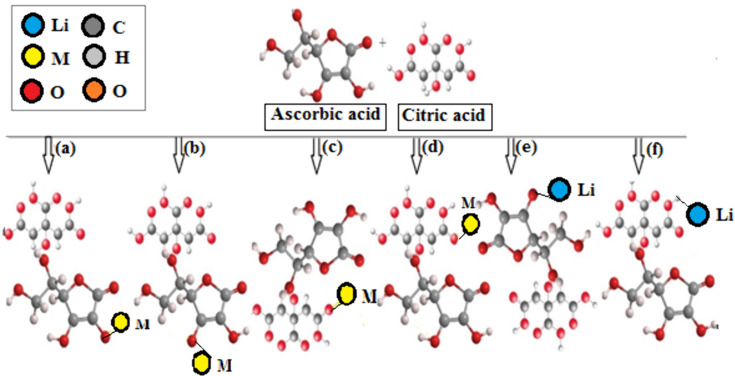
Possible metal complexes with mixed organic acids and coir peat.

Ascorbic acid can act as a reducing reagent through supplying the oxidized metal compounds in the sample with an electron. By double oxidation of ascorbic acid (C_6_H_8_O_6_) to its radical form (C_6_H_7_O_6_), it obtains the stable form of dehydroascorbic acid (C_6_H_6_O_6_). The metal ions in the sample with a higher oxidation state are simultaneously turned into a lower oxidation state. Alkenes, alcohols and ester functional groups present in ascorbic acid system, are groups in which the conjugated systems are formed mainly by OH lone pair and C–C and C–O electrons. The resonating structures which form very stable deprotonated conjugate base, combine with valuable metals released from spent cathode material to form complexes [23]. Some possible acid complexes are shown in Fig 3.

The bulk of the methoxy and free hydroxyl groups in coir peat (cellulose) contains many primary and secondary hydroxyl groups, having a network–like structure. Such chemical functionalities adsorb a variety of important metals, even dilute acids completely desorb them, indicating that this adsorption is of an ionic character (Equation 8).


$$2Coirpeat–COONa+M^{+2}\to (Coir–COO{)}_{2}M+{2Na}^{+}\mathrm{\backslash ]}$$ (8)

**Fig 4:**
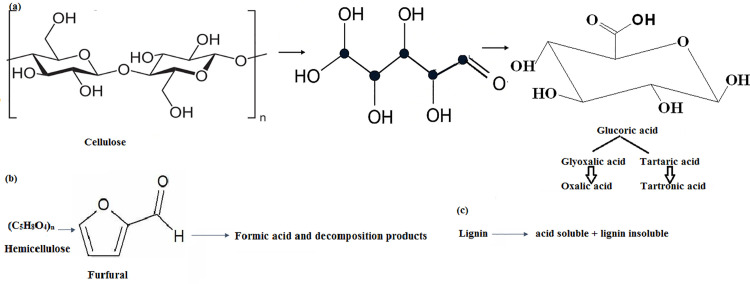
Possible reducing agents of coir peat (a) Cellulose into Glucoric acid (b) Hemicellulose into formic acid and decomposition products (c) Lignin into soluble acids.

By increasing the contact surface, porous coir peat can be used as a reductant to accelerate the adsorption of acidic and cathode materials. Additionally, hydrolysis products of coir peat can be used as an environmentally friendly substrate to lower high valence metal ions [24]. Consequently, coir peat surface is enriched with electron–donating oxygen functionalities as shown in Fig 4.

Coir peat lignocellulosic content was measured using, following formula, to determine their cellulose content.


Cellulose%=100%−hemicellulose%−lignin%−ash%−extractive%\] (9)

The CHNS elemental analyzer was used to determine the absolute elemental composition. The following formula determined the content percentage of oxygen:


Oxygen%=100%−C%−H%−N%−S%ash%\] (10)

Organic acids (AA–CA) may solvate and remove metals from the battery cathode material, which contains metal oxides or salts. They are mostly utilized to leach metal ions from used LIBs. By rupturing the metal–oxide bonds in the used battery materials, ascorbic acid (C_6_H_8_O_6_) and citric acid (C₆H₈O₇) may dissolve metal ions like nickel (Ni²⁺), cobalt (Co²⁺), and lithium (Li⁺) and generate soluble metal complexes as shown in Equation (11–14). Once they are discharged into solution, coir peat (a natural adsorbent) can bind to metal ions and cause adsorption due to the presence of a variety of functional groups, like carboxyl (–COOH) and hydroxyl (–OH) functional groups.

Adsorption occurs when metal ions from solution are drawn to coir peat surface by hydrogen bonds, electrostatic interactions, or coordination with functional groups (such as Co^2^ ⁺ , Ni^2^ ⁺ , etc.) without causing a substantial change in the metals’ valence state. To improve its ion–exchanging properties, it can also be altered or treated with certain organic acids. The process may enable the coir peat to exchange protons (H^+^) or other ions with metal ions, for example nickel, cobalt, and lithium ions in a selective manner. When subjected to acidic conditions in the leaching solution, cobalt (Co³⁺) or nickel (Ni³⁺) may be reduced to a lower oxidation state (Co²⁺ or Ni²⁺).

**1-Citric acid-metal ion chelation**


$${{Co}^{+2}+C_{6}H_{8}O_{7}\to \left[{Co\left(C_{6}H_{8}O_{7}\right)}_{2}\right]}^{-2}\mathrm{\backslash ]}$$ (11)

Citric acid chelates metal ions, like Co^+2^, Ni^+2^ forming stable complexes.

**2-Ascorbic acid-metal ion reduction**


$${Co}^{+3}+C_{6}H_{8}O_{6}\to {Co}^{+2}+C_{6}H_{6}O_{6}+{2H}^{+}\mathrm{\backslash ]}$$ (12)

Ascorbic acid reduces high-valent metal ion Co^+3^ to low-valent ions Co^+2^

**3-Adsorption on coir peat**


$${Co}^{2+}+Coirpeat(surface-COOH)\to Coirpeat\left(surface-COO-Co^{2+}\right)\mathrm{\backslash ]}$$ (13)

Metal ions are adsorbed onto the surface of coir peat.

**4-Ion exchange reaction with coir peat**


$$Coirpeat(surface-COO-H)+{Li}^{+}\to Coirpeat(surface-COO-Li)+H^{+}\mathrm{\backslash ]}$$ (14)

Ion exchange of Li^+^ with proton (H^+^) from coir peat surface.

## Results and discussion

### Optimization of leaching system

#### Impact of coir peat with mild organic acid.

**Fig 5:**
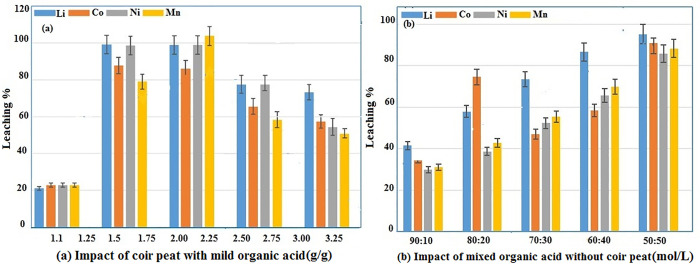
(a) Impact of coir peat and mild organic acid, (b) Impact of mild organic acid without coir peat.

Significance of coir peat dosage on leaching efficiency was examined under same circumstances such as 55 ˚C, 460 rpm of agitation speed, reaction time of 55 minutes, solid–liquid ratio of 20 g/L and mixed acid (citric and ascorbic acid) concentration of (50:50 mol/L). As shown in Fig 5a, increasing the coir peat concentration (1, 1.25, 1.5, 1.75, 2.00, 2.25, and 2.50 g/g) in triplicate (n = 3) to ensure, accuracy variations in metal recovery were generally below ±2%, confirming the reliability of the data, results in an increase in leaching efficiency of Li, Co, Ni, and Mn 98%, 84.6%, 85.6%, and 79.8%, respectively. Leaching efficiency slightly rises when the concentration of coir peat is increased from 1.5 g/g to 2.25 g/g. However, when dose of coir peat is greater than 2.25 g/g, the leaching efficiency falls. The decrease in efficiency beyond 2.25 g/g is attributed to adsorption of metal ions onto the coir peat, because more coir peat on the surface area is available to bind metal ions, this cause dissolved metals to reattach to solid phase rather than remain in the solution, reducing the leaching efficiency. SEM analysis of the post-leaching residue showed morphological changes and surface deposition consistent with metal ion binding. Additionally, ICP analysis confirmed the presence of residual metal ions on the solid phase after leaching. Coir peat can accelerate reactions by reducing the transitioning between high and low valence metals. Excess of coir peat, however, may adsorb certain metal ions and lessen the leaching efficiency.

Oxidized coir peat demonstrated superior metal leaching efficiency relative to unmodified coir peat, presumably owing to its augmented surface area and elevated concentration of functional groups, which facilitate metal complexation and acid interaction. This increase proves the idea that oxidation treatment of coir peat can markedly boost the efficacy of the leaching process. In this way, 1.5 g/g of coir peat was used as optimal coir dosage.

#### Impact of mixed organic acid concentration without coir peat.

A major influence on the leaching process is the total concentration of mixed organic acid. By keeping other factors constant, such as temperature of 55 ˚C, agitation speed of 460 rpm, reaction time of 55 minutes, without coir peat, and solid to liquid ratio of 20 g/L, effect of variation in the mixed acid (citric and ascorbic acid) concentration (90:10, 80:20, 70:30, 60:40, 50:50 mol/L) was investigated. Compared to previous reactions which have a coir peat as a reductant, using AA–CA have less leaching efficiency, acting as a controlled reaction. According to Fig 5b, leaching efficiencies increased as total acid concentration rises, reaching 97.60% for Li, 85.56% for Co, 83.06% for Ni, and 84.11% for Mn at 50:50 mol/L. However, efficiency of leaching for Li, Co, Ni, and Mn stayed almost persistent when concentration was raised to 50:50 mol/L, probably due to attaining the point of equilibrium that is the dissociation of H^+^ ions in citric and ascorbic acid system. It was concluded that 50:50 mol/L was the ideal concentration of mixed acid without significantly increasing reagent consumption, based on a balance between metal efficiency and practical considerations such as reagent availability, cost, and ease of preparation. Additionally, by avoiding the overuse of either acid, this ratio makes the process more cost-effective and scalable.

### Effect of temperature

**Fig 6:**
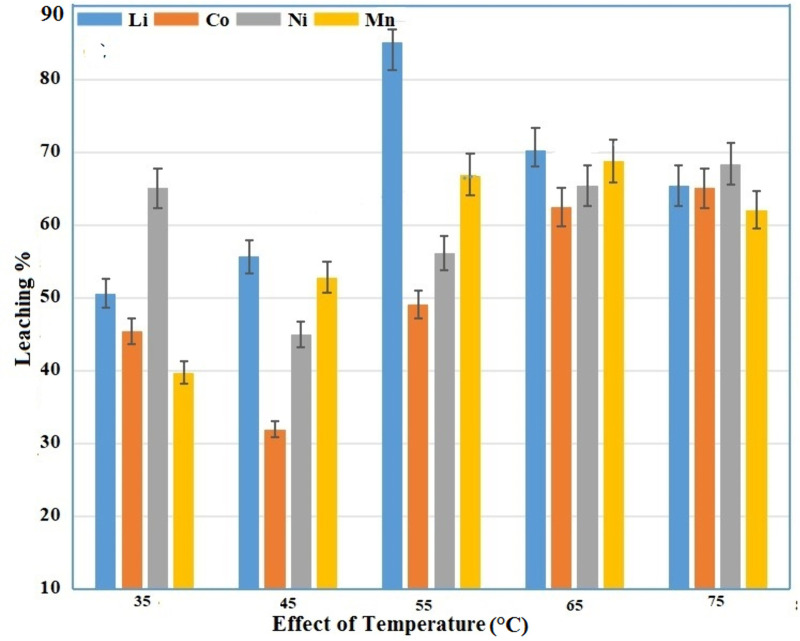
Effect of temperature under different leaching conditions.

Leaching efficiencies were influenced by reaction temperature. Fig 6 shows the effect of temperature between 35 °C to 75 °C, at a stirring speed of 460 rpm, 1.5 g/g of coir peat, concentration of 50:50 mol/L ascorbic and citric acid, 20 g/L solid to liquid ratio and 55 minutes of the reaction time. Leaching efficiency improved with rising the reaction temperature. It makes sense that increasing the reaction temperature (55 °C being the optimal), accelerates the rate of leaching reaction. Hence, leaching efficiency could be increased at a certain time before complete leaching due to higher leaching reaction rate. Leaching process of spent LIBs uses a standard endothermic reaction to recover metals from cathode materials [25,26]. Leaching efficiency as well as reaction rate, therefore increases with temperature.

### Effect of time

**Fig 7:**
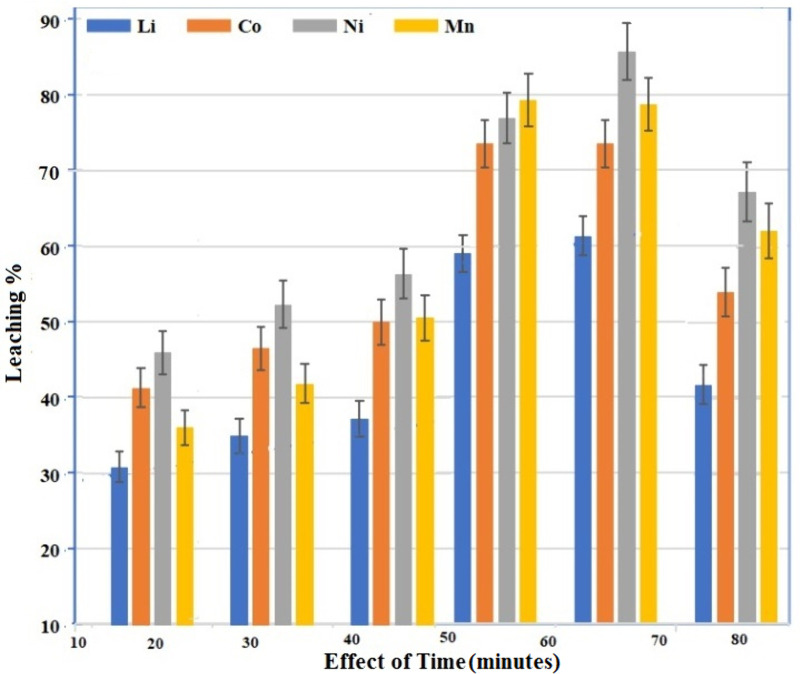
Effect of time under different leaching conditions.

Keeping all other experimental parameters constant, the impact of leaching time of more than 80 minutes was examined with stirring speed of 460 rpm, a concentration of 50:50 mol/L mixture of ascorbic and citric acid, 1.5 g/g of coir peat, solid to liquid ratio was 20 g/L, and 55 °C temperature. The results shown in Fig 7, clearly define the impact of duration of contact between solid and liquid phases on leaching efficiency. It was found that the leaching rate was high, especially in the early phase of the process as up to 40% Li, 45% Co, 60% Ni and 50% Mn dissolved in the first fifteen minutes and the effectiveness considerably increased until about 55 minutes (Li: 59%, Co: 74%, Ni: 78%, and Mn: 79%). Because ascorbic acid and citric acid reduced in action to facilitate easier dissolution in leaching solution, higher value metals are converted to their reduced state, which results in comparatively low leaching of the cobalt and lithium at primary stage [27]. Longer leaching (more than 70 minutes) showed a decrease in efficiency. This may be attributed to the decomposition of ascorbic acid and citric acid upon prolonged contact with cathode material even at some ambient conditions.

### Effect of agitation speed

**Fig 8:**
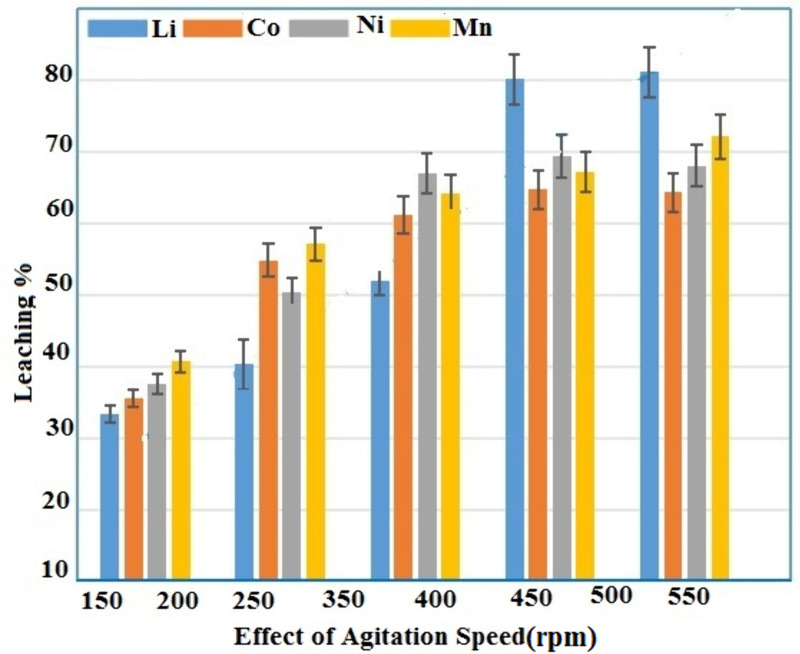
Effect of agitation speed under different leaching conditions.

In heterogeneous processes, the speed of stirring is crucial to accelerate the leaching process in lixiviants while stimulating solid–liquid phase by visible mass transfer. To find out how the cathode material’s agitation speed affected the leaching system of metals, the solution was stirred from 150–550 rpm. Other parameters stayed the same, including time of 55 minutes, 55 ˚C of temperature, solid–liquid ratio of 20 g/L, 1.5 g/g of the coir peat, and aqueous mixture of organic acid concentration (50:50 m/L ascorbic and citric acid). The findings are displayed in Fig 8 which demonstrates that as the stirring speed increased, it correspondingly increased the leaching efficiency of valuable metals [28] with 460 rpm selected as the optimum agitation speed.

### Effect of solid to liquid ratio

**Fig 9:**
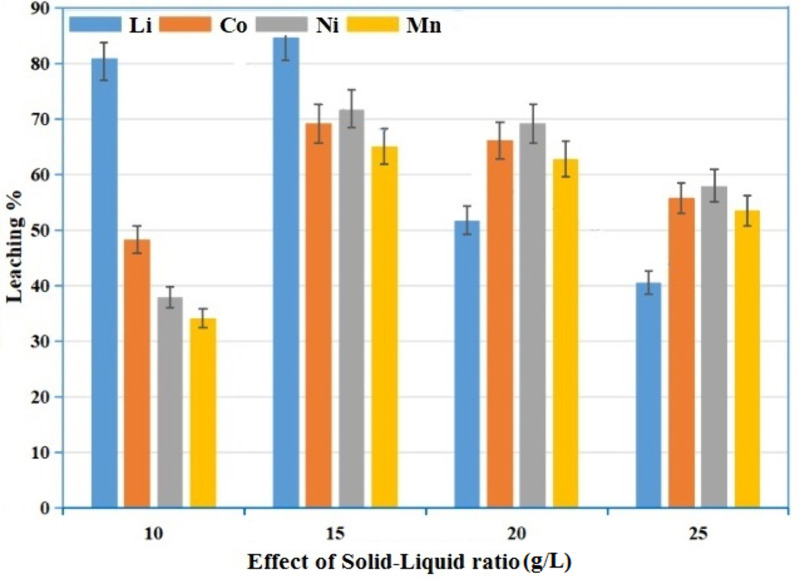
Effect of solid to liquid ratio under different leaching conditions.

Fig 9 shows the effect of solid liquid ratio on leaching performance with an optimal solid–liquid ratio between 15 g/L and 20 g/L. The impact of fluctuations on the solid liquid ratio (g/L) was investigated whereas all other parameters were set at 55 °C, a stirring speed of 460 rpm, 1.5 g/g of coir peat, a concentration of 50:50 mol/L water mixture of ascorbic acid and citric acid, and a reaction time of 55 minutes. The leaching efficiency increases with an increase in solid liquid ratio with 20 g/L is the best ratio and decreased at higher ratios [29].

### Kinetics leaching of valuable metals (Li, Co, Ni, and Mn)

Leaching kinetics and mechanism were observed by analyzing data at various temperatures (T1, T2, T3) with respect to time. Assuming small particles of cathode powder, the collected data were analyzed to match the standard models of heterogeneous processes, such as shrinking core models (SCM) Equation 15 and diffusion–controlled models Equation 16 with chemical control mechanism:


$${1-(1-x)}^{\frac{1}{3}}=k_{c}t\mathrm{\backslash ]}$$ (15)

$${1-(1-x)}^{\frac{2}{3}}=k_{d}t\mathrm{\backslash ]}$$ (16)

t = time fraction (in minutes),

x  = reactant’s mass transfer rate

k_c_ and k_d_ = rate constants

The kinetic data did not align well with equations (15) and (16) (which are not shown for brevity). Consequently, the following Equation 17 were employed to assess empirical model [30,31].


$${\left\{-\ln(1-x)\right\}}_{2}=k_{emp}t\mathrm{\backslash ]}$$ (17)

$$K={Ae}^{-\frac{Ea}{RT}}$$ (18)

Where A and T represent the Arrhenius constant and temperature (in kelvins), respectively.

k = reaction constant (min^−1^)

*Ea* = activation energy of metals (Li, Co, Ni, and Mn)

R = universal constant of gass (8.31 J/K/mol)

The rate constants for the leaching reactions were obtained by fitting the data to appropriate models, leaching efficiency data were plotted against time, and the rate constant (k) was calculated from the slope of the linearized form of the kinetic model. The fitting was performed using linear regression analysis, and goodness of fit was evaluated to confirm the models suitability. All calculations followed standard methods as reported in [32].

**Fig 10:**
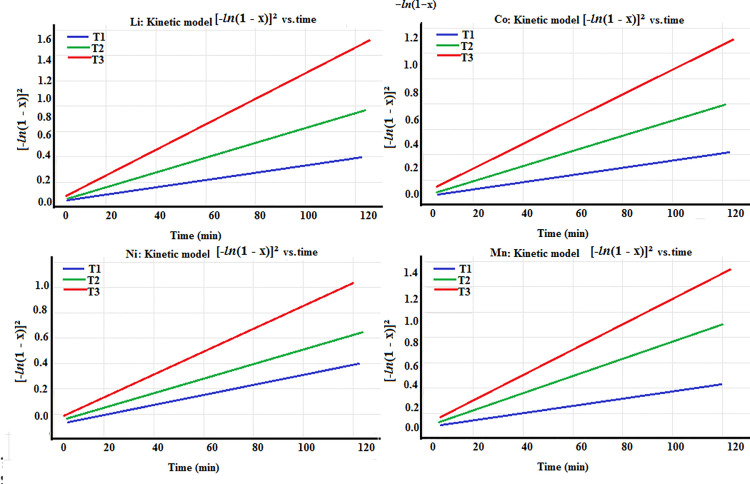
Kinetic analysis [−*In*(1 − x)]^2^ vs. time at various temperature for valuable metals Li, Co, Ni and Mn.

Fig 10 shows the kinetic analysis of empirical model; linear lines confirms the stability of model. As temperature increases from T1 to T3, slopes become steeper, increases high reaction rates.

*Ea* provides an insight into the chemical nature of rate–limiting step. Total reaction rate is frequently regulated by the rate–limiting step in chemically controlled processes. For instance, in metal leaching, dissolution of most metal ions from cathode material, is perhaps the rate–determining step, whereas in adsorption or ion exchange, the metal ions’ chemical binding to the adsorbent becomes the rate determining step. Chemically controlled mechanism follows a second–order kinetic model, which reflects the exchange of ions between the solution and the adsorbent:


$$\frac{d[C]}{dt}=k_{2}[C]\left[H^{+}\right]$$ (19)

C is the concentration of metal ion in solutions and [H⁺] is the concentration of protons exchanged from the adsorbent.

**Fig 11:**
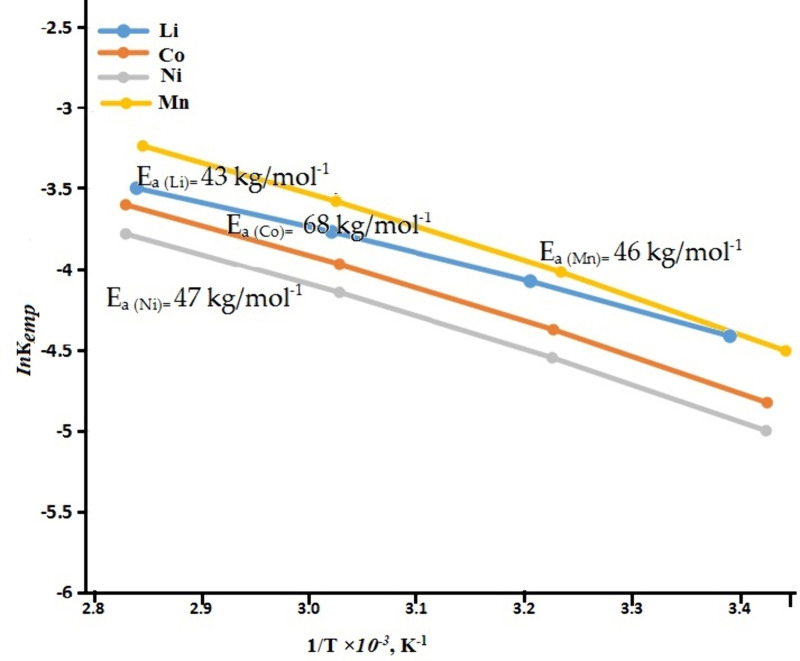
Arrhenius plot at different temperatures for metal leaching.

Fig 11 displays plots of *ln*k_emp_ vs. 1/T × 10^−3^, K^-1^ generated for metals such as Li, Co, Ni, and Mn. Apparent activation energy (*Ea*) for each valuable metal was then computed using the slope values that were found from the plots’ straight lines. The *Ea* values obtained were 43 kJ mol^−1^ for Li, 68 kJ mol^−1^ for Co, 47.8 kJ mol^−1^ for Ni and 46 kJ mol^−1^ for Mn, demonstrating unequivocally that the chemically controlled mechanism is responsible for the metals’ dissolution process. The higher *Ea* value of cobalt could be attributed to the two distinct oxidation states of Co(II) and Co(III) [33]. Overall quantitative leaching of the valuable metal requires the conversion of cobalt (III) to cobalt (II), which needs more energy to defeat the energy block in a solution of mixed organic acid to dissolve the metal ions [34].

### SEM analysis

**Fig 12:**
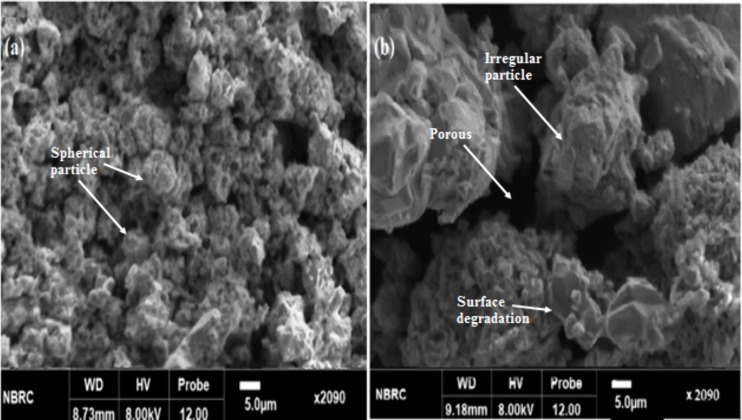
The scanning electron microscopic images (a) residue before leaching (b) residue remaining after leaching treatment using mixed organic acids and coir peat.

SEM was used to view cathode black powder’s morphology. Powder was made up of uniformly sized particles that were dispersed having an average size of 6 μm (size range was from 1 to 12 μm). The comparison between cathode leaching with a combination of organic acids and coir peat shows that the particles were clumped together with a nearly spherical shape and that there was a complete change from spherical to irregular particle size, which was consistent with leaching results by using coir peat. This might be attributed to the precipitates of metals that were leached from the cathode powder and were subsequently redeposited during the operation. The cathode material was degraded by organic acids and coir peat, as evidenced by particle size and shape before and after leaching. The development of fresh deposits or crystalline structures on the cathode material were shown in Fig 12a which is produced through stacking materials in the form of sheets, is incredibly smooth.

This process breaks down the initial structure of metal oxide when it encounters ascorbic acid, citric acid, and coir peat in the leaching solution. Finally, the solution is filled with metal ions. As shown in Fig 12b, due to fibrous and porous nature, the coir peat may show surface changes, such as increased porosity or decomposition, which suggests that it, has encountered the acids and cathode powder. The breakdown of cellulosic component of coir is caused by oxidation, which results in chain scission as well as the formation of carboxyl groups. Upon oxidation, lignin component produces dicarboxylic acids that are soluble in water. In general, oxidation caused coir to lose strength, but not enough to change the material’s physical (fibrous) shape. It was noted that leaching residue has more holes and defects, which suggests that the substance was corroded by mixed acids with coir peat.

SEM pictures of the material before and after leaching are displayed in Fig 12a and 12b. Particles first seem to be mostly spherical and have smooth surfaces in before leeching. Surface was compact and covered with binder which limited leachant penetration. The attack of bio metabolites, especially organic acids, led to metal dissolution [35]. Effective metal extraction is indicated by the particles’ uneven morphologies, increased porosity, and apparent surface damage during leaching.

### X–ray diffraction analysis

**Fig 13:**
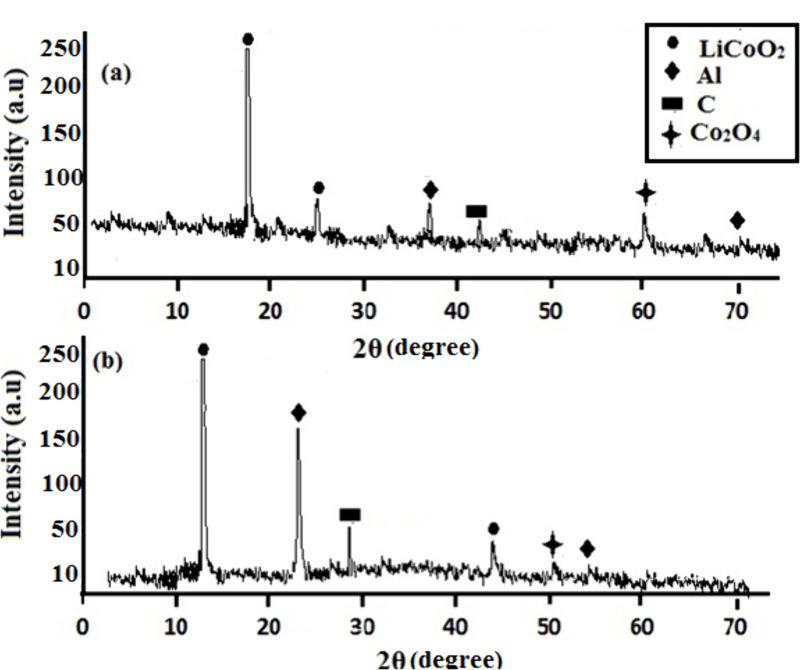
XRD patterns of (a) cathode powder before leaching (b) and residue after leaching.

A representative X–ray diffractogram of wasted Li–ion battery cathodes, both before and after leaching with organic acid and coir peat is displayed in Fig 13, indicating the presence of metals in the structure. Leaching of cathode powder in mixed organic acids (AA–CA) without reducing agent does not substantially change the XRD’s distinctive peaks. This is due to the extremely poor metal recovery after leaching, except for lithium, which had a comparatively better recovery. After leaching with coir peat as reducing agent, the cathode samples included LiCoO_2_, Co_3_O_4_, Al, and carbon. After removing the binder, 2θ scattering of Al at 37˚ was shifted to 22˚. A couple of additional two patterns and intensities, 61˚and 19˚, were also shifted to 50˚ and 12˚. The possible cause could be the defects that emerged from removing the binder.

LiCoO₂ at around 2θ ≈ 19°, 25°, Al at 2θ ≈ 37° and 70° and C peak 2θ ≈ 42° were higher in Fig 13(a), suggesting that the sample has better crystallinity of these phases with high intensities [36]. The sample has undergone some treatment of leaching with organic acids, that decreased the crystallinity of phases, especially LiCoO₂, around at 2θ ≈ 10°, Al at 2θ ≈ 22° and 52° have lower intensity and C peak at 2θ ≈ 27° as indicated by the lower overall intensities (Fig 13b). By adding more acids dissolved Co₂O₄ during the process, as evidenced by its presence in Fig 13a but absence or very slight presence in Fig 13b. Even after the addition of reductant (coir powder), the Co_2_O_4_ phase in the residual structure remained intact, demonstrating its strong resistance to dissolution. It shows that, even in the presence of a reducing agent, it had become resistant to dissolving in organic acid (AA–CA).

### FT–IR analysis

**Fig 14:**
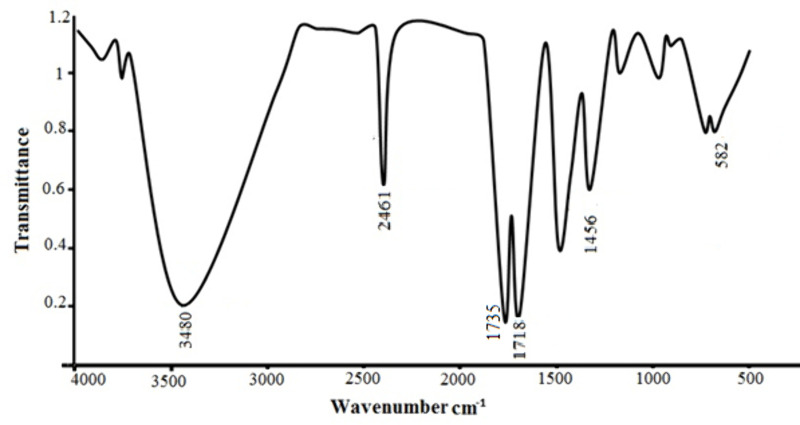
FTIR spectra of residue left after leaching with mixed acids and coir peat.

**Fig 15:**
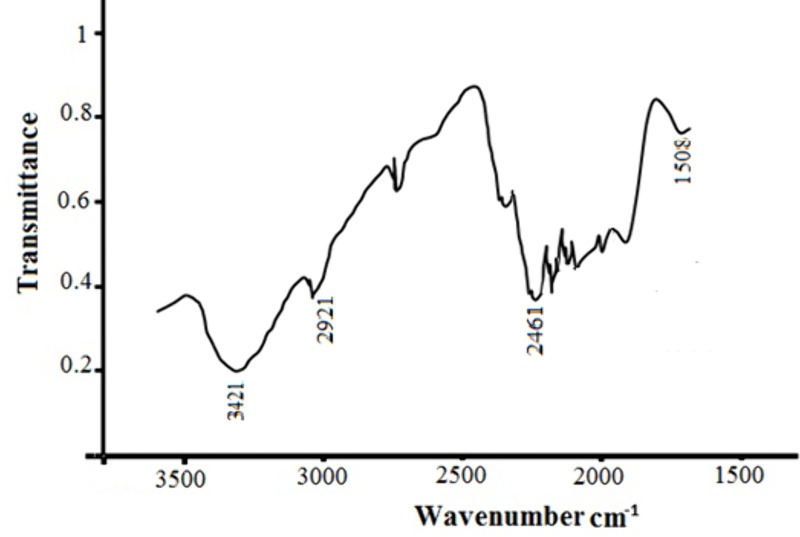
Unmodified Coir-peat.

As shown in Fig 14, changes in the functional groups of residue, were analyzed after leaching with mild acid and coir peat. The entire spectrum between 4000 cm^–1^ and 400 cm^–1^ was split into characteristic as well as fingerprint regions. Additionally, the peaks at 582 cm^−1^ belong to the bond coordination reactions between metal ions and ligands. The functional group and chemical bonds of organics can be determined using the characteristic region. Oftentimes, the aromatic rings C = C stretching in lignin, is responsible for the 1456 cm ⁻ ^1^ peak. Band at 1718 cm^–1^ has been given to CO stretching, associated with carbonyl groups, mainly from lignin and hemicellulose. A strong peak around 1718 cm ⁻ ¹ showed the C = O stretching of mixed organic acids. An extra peak, which is indicative of the carboxyl group (COOH), was seen at 1735 cm^−1^ after oxidation. Because more pores open up in oxidized coir peat than in unmodified coir peat, which has nearly no holes, oxidized coir peat is more accessible. The IR plot of unaltered coir fibers shown in Fig 15.

Rarely, weak absorption bands around 2461 cm ⁻ ¹ seen in atmospheric CO_2_, if it is present in the sample or the surrounding air at the time. The broad peak around 3480 cm^⁻1^, hydroxyl groups present in cellulose and lignin, confirmed the result of their presence. The present study’s FT-IR spectra closely matched those of previous studies [37,38].

### UV–Visible analysis

**Fig 16:**
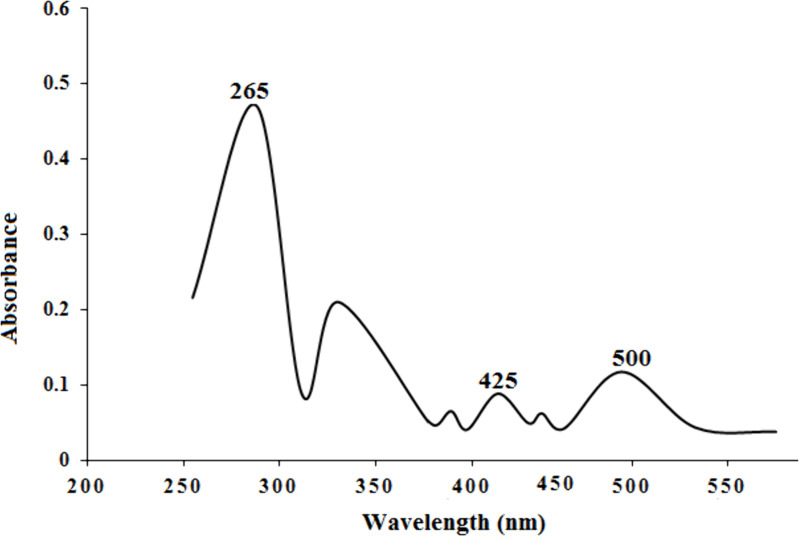
UV–visible spectrum of the leachant.

As shown in Fig 16, mixed organic acids and their anions are responsible for the broad and very strong absorption peak at 227 nm. Citric acid showed a weak absorption in the far UV region, while a distinct peak around 265 nm has been assigned to ascorbic acid. Cobalt complexes formed during the process of leaching are indicated by the absorbance peak around 500 nm due to d–d transition, an evidence of Co (II) released from the oxide lattice through M–L complexation with citric and ascorbic acid [39]. As the leaching increases, the absorption peaks seem to rise. The complexation of M (Li, Co, Ni, Mn) ions with the ligand (L) would become the cause of increase in peak absorption. The peaks around 425 nm to 450 nm belong to Mn–L and Ni–L complexes. Moreover, the spectra make it evident that the presence of the broad peak absorption of coir peat, as a good reducing agent, in leaching solution causes the reduction of M(III)–L to M(II)–L with UV range of 250 nm to 300 nm. As far as our knowledge goes, neither Mn nor Ni–complexes have any reports accessible.

### Recovery of valuable metals

Solvent extraction and precipitation techniques were used to recover Mn, Ni, Co and Li by leaching cathode powder in mixed organic acid and coir peat solution from wasted LIBs. First, Ni^2+^ was selectively precipitated through one of the selective organic agent “dimethylglyixime,” with molecular formula C_4_H_8_N_2_O_2_ that is soluble in CH_3_OH or in NaOH solution. At equilibrium conditions, 98% of Ni could be recovered as precipitate of nickel dimethylglyixime chelating compound.


$$Recovery\%=\frac{C_{i}V_{i}\times {C_{f}V}_{f}}{C_{i}V_{i}}\times 100\mathrm{\backslash ]}$$ (20)

C_i_ showed the initial concentration of metal (mg/L), V_i_ initial volume of solution (L) and C_f_ showed the final concentration of metal after recovery (mg/L), and V_f_ as final volume of solution (L) shown in Equation 20.

During the precipitate’s dissolution in 1 mol/L HCl solution in C_4_H_8_N_2_O_2_, Ni and other components could be separated from one another. Dimethylglyoxime as a precipitant can be renewed and reutilized. Ammonium oxalate reacts to produce insoluble metal oxalates with metal ions. When ammonium oxalate is added to a solution containing cobalt ion (Co²⁺), it forms cobalt oxalate [CoC₂O₄] precipitate [40,41].

Mn^2+^ can form complex compounds with citric acid and ascorbic acid, making precipitation problematic. Citric acid has poorer chelation with Co^2+^, allowing it to react with C_2_O_4_^–2^ and precipitate as [CoC_2_O_4_]. Furthermore, co–precipitated [MnC_2_O_4_] can get dissolved in a dilute solution of oxalic acid. Lithium was precipitated as [Li_3_PO_4_] using 0.4 mol/L solution of Na_3_PO_4_, with an approximate recovery of 89%.

Di-(2-ethylhexyl) phosphoric acid (D2EHPA), an organo phosphorus compound is used for solvent extraction of valuable metals [42,43]. Manganese was effectively extracted using 20 vol% of D2EHPA at 460 rpm of agitation, 50:50 mol/L ratio of mixed organic acid concentration, 1.5 g/g of coir peat, temperature 55 °C, retention period 55 min, and solid to liquid ratio was 20 g/L. 99% of the Mn^2⁺^ was recovered by stripping 0.1 mol/L H₂SO₄, and D2EHPA was regenerated for further usage (Table 2).

**Table 2:** Recovery percentages of various metals (Ni, Co, Mn, and Li) from spent lithium-ion batteries using precipitation and solvent extraction methods*.*

Sr. No	Metal	Recovery Method	Extracting Agent	Conditions	Recovery %
1	Nickel (Ni²⁺)	Selective Precipitation	Dimethylglyoxime (C₄H₈N₂O₂)	Methanol/NaOH, equilibrium conditions, precipitation + HCl dissolution	98%
2	Cobalt (Co²⁺)	Precipitation	Ammonium Oxalate (NH₄)₂C₂O₄	Selective reaction with Co² ⁺ , forming CoC₂O₄	Not specified
3	Manganese (Mn²⁺)	Solvent Extraction + Stripping	D2EHPA (20 vol.%) + 0.1 mol/L H₂SO₄	460 rpm, 55°C, 55 min, 50:50 mol/L organic acid, 1.5 g/g coir peat, solid:liquid = 20 g/L	99%
4	Manganese (Mn²⁺)	Co-precipitation	Citric/Ascorbic Acid + Oxalic Acid	MnC₂O₄ can dissolve in dilute oxalic acid, affecting precipitation	Variable
5	Lithium (Li⁺)	Precipitation	Na₃PO₄ (0.4 mol/L)	Precipitation as Li₃PO₄	89%

## Conclusions

The study showed that coir peat combined with ascorbic acid and citric acids can effectively stimulate leaching process of valuable metals including Li, Co, Ni and Mn of spent lithium ion batteries (LIBs) without adding chemical reducing agents. At optimal conditions such as stirring speed of 460 rpm, mixed organic acids concentration of 50:50 mol/L, temperature of 55 °C, retention time period of 55 minutes, 1.5 g/g of coir peat, and solid to liquid ratio of 20 g/L, a high leaching efficiency was achieved for Li, Co, Ni, and Mn (98%, 84.6%, 85.6%, and 79.8%, respectively). A chemically controlled mechanism was discovered that directs the entire process. By enhancing stability and control of leaching environment, coir peat can improve extraction of metals from wasted LIBs cathode powder in hydro–metallurgical operations. Kinetic analysis revealed that organic acid mixtures reduce acid demand, while hydrolyzed coir peat releases natural reducing agents that enhanced leaching efficiency. Instead of using harsh and dangerous mineral acids, the current study uses a biodegradable coir peat to advance sustainable metallurgy. The activation energy values of lithium, cobalt, nickel, and manganese were calculated to be 43, 68, 47 and 46 kj/mol, respectively. The results showed that using a combination of mixed organic acids (ascorbic–citric acid) as novel and eco–friendly green reagents and coir peat as environmentally benign, to recycle valuable metals from different waste batteries (including electronic scrap), can be a much profitable approach in recycling.
